# Phylogenetic studies on three *Helicotylenchus* species based on 28S rDNA and mtCOI sequence data

**DOI:** 10.21307/jofnem-2019-033

**Published:** 2019-06-06

**Authors:** K. Rybarczyk-Mydłowska, E. Dmowska, K. Kowalewska

**Affiliations:** 1 Museum and Institute of Zoology PAS, Wilcza 64, 00-679, Warsaw, Poland

**Keywords:** Cytochrome c oxidase subunit I, Genetic code alterations, *Helicotylenchus canadensis*, *H. pseudorobustus*, *H. varicaudatus*, Ribosomal DNA, Spiral nematodes

## Abstract

To facilitate the process of spiral nematode species delineation, populations of *Helicotylenchus canadensis*, *H. pseudorobustus*, and *H. varicaudatus* deriving from various locations and diverse natural and anthropogenic environments from Poland were investigated and characterized. For the first time, 28S rDNA sequences are reported for *H. canadensis* and *H. varicaudatus*, whereas new mtCOI sequences were acquired for all three analyzed species. A Bayesian phylogenetic analysis of the 28S rDNA fragments revealed that *H. canadensis* and *H. varicaudatus* are members of a clade that is a sister group to all other *Helicotylenchus* species; however, the closest known sister group to *H. canadensis* is *H. vulgaris* type A. Both 28S rDNA- and mtCOI-based phylogenetic results suggest that this clade excludes *H. pseudorobustus*, whose most recent common ancestor with the former species was inferred to be the ancestor of all *Helicotylenchus* species. Moreover, within the mtCOI sequences obtained from *H. pseudorobustus*, unlike from the other two, a simultaneous presence of TAG and TAA codons was identified. This may indicate mitochondrial genetic code alterations or other genomic rearrangements in *H. pseudorobustus*.

Spiral nematodes, which belong to the *Helicotylenchus* spp. (Hoplolaimidae), are representatives of Tylenchomorpha, which is considered the most successful lineage of plant parasitic nematodes. They evolved by gradual transitions from fungal feeders through facultative plant parasites toward obligatory plant parasites (Holterman et al., 2017). Furthermore, the phylogenetic tree of this group reveals main evolutionary pathways, leading from ectoparasitism or migratory endoparasitism to the most specialized, sedentary endoparasitic lifestyles, exhibited by the most harmful plant-damaging nematode species. Although the majority of research focuses on the latter pests, the investigation of species that developed less sophisticated mechanisms of parasitism should not be overlooked. Analyses of molecular information hidden in their genomes bring us closer to an explanation why some groups of nematodes became more successfully adapted to plant parasitism than others.

Unlike the closely related, sedentary endoparasitic cyst nematodes, most representatives of the genus *Helicotylenchus* (Steiner, 1945) are considered mild plant pathogens of minor economic importance. However, several species like *H. dihystera* (Cobb, 1893; Sher, 1961), *H. digonicus* (Perry in Perry, Darling & Thorne, 1959), *H. multicinctus* (Cobb, 1893; Golden, 1956), *H. pseudorobustus* (Steiner, 1914; Golden, 1956), or *H. varicaudatus* (Yuen, 1964) were proven to be associated with plant growth suppression or more severe crop-plant damage (Perry et al., 1959; Siddiqi, 2000; Schreck Reis et al., 2010). They are classified as ectoparasites or semi-endoparasites and can be found in root systems of diverse cultivated and uncultivated plants (Siddiqi, 2000; Subbotin et al., 2011). In total, this cosmopolitan genus encompasses over 200 described species (Uzma et al., 2015), of which many await a more detailed characterization.

To determine particular *Helicotylenchus* species, a precise evaluation of morphological and morphometrical features is used, usually after the preliminary recognition of the characteristic coiled body shape observed in the relaxed state or after death. However, the available keys do not always allow for proper species identification due to high intra-specific and minor inter-specific variability within the genus (Fortuner, 1984; Fortuner et al., 1984). The proposed diagnostic characters and features can match and overlap between closely related species. Therefore, to facilitate the species identification process as well as to delineate more phylogenetically distant species, which share the same morphology (cryptic species), the support of a molecular approach is needed.

To date, only about 20% of *Helicotylenchus* species has been molecularly characterized using mostly ribosomal DNA fragments (18S, ITS, 28S rDNA; GenBank resources). Mitochondrial cytochrome c oxidase subunit I (mtCOI) sequences were reported for one species, the recently described *H. oleae* (Palomares-Rius et al., 2018), and a cytochrome c oxidase subunit II sequence was reported solely for *H. dihystera* (Riepsamen et al., 2011). A combination of both the nuclear genome-derived large subunit ribosomal DNA gene (28S rDNA) and more variable genes such as mtCOI has been suggested to comprise valuable markers for subsequent phylogenetic analyses in this group (Subbotin et al., 2011; Palomares-Rius et al., 2018).

The objectives of this study were as follows: to deliver molecular characteristics of three *Hecicotylenchus* species commonly occurring in Poland, namely, *H. canadensis*, *H. pseudorobustus* and *H. varicaudatus*, by the use of nuclear 28S rDNA and mtCOI data, and to further evaluate the potential of mtCOI sequences for species identification and phylogenetic study of the genus *Helicotylenchus.*

## Materials and methods

### Sampling, nematode extraction, and conservation

Three Hoplolaimidae species originating from Poland were analyzed: *H. canadensis*, *H. pseudorobustus*, and *H. varicaudatus*. Nematodes were collected during a study conducted between 2010 and 2014 on the characterization, occurrence, and distribution of plant parasitic nematodes in Poland. Soil samples were derived from various habitats and vegetation types. Each sample (about 1 kg of soil) was taken to a depth of 30 cm from the root zone using a soil sampler. Nematode extraction was performed by the decantation and sieving method, followed by the centrifugal flotation method (van Bezooijen, 2006). Nematodes were killed with hot water at 60°C. The parts of the samples of selected Hoplolaimidae were fixed in TAF and designated for morphological analysis. The rest of the samples were fixed in DESS and given to molecular studies.

### Morphological identification

Morphological observations and morphometrical analyses were performed using Leica light microscope with Nomarski differential interference contrast. Morphological identification was performed using identification keys and descriptions by Waseem (1961), Yuen (1964), Sher (1966), Brzeski (1998), and Andrássy (2007). Nematodes fixed in DESS were subjected to morphological vouchering and DNA amplification procedure of Yoder et al. (2006). Nematodes were identified on the temporary slides, and subsequently multifocal images were made for every specimen.

### DNA extraction

After morphological identification, analyzed nematode individuals were marked with specific codes and assigned for further molecular studies. Genomic DNA from single nematode specimens was extracted using either GenElute™ Mammalian Genomic DNA Miniprep Kit (Sigma-Aldrich) or QIAamp DNA Micro Kit (Qiagen) according to the manufacturers’ instructions. For each sample, DNA was eluted in 30 μl H_2_O. Extracted DNA was stored at −20°C.

### Primers, DNA amplification, and sequencing

The mitochondrial DNA fragments of the mtCOI as well as the genomic DNA fragments of the large subunit rDNA (28S rDNA) were amplified from the collected Hoplolaimidae species. Initially, during the ongoing survey project, the publicly available JB3 and JB4 or JB5 mtCOI primers (Hu et al., 2002; Derycke et al., 2010) were tested for amplification of mtCOI fragments from numerous plant parasitic species. However, from these primers, PCR amplification failed. Therefore, a new set of primers was developed according to the slightly modified primer design methodology, as in Rybarczyk-Mydłowska et al. (2012, 2014). An alignment comprising of mtCOI publicly available sequences from different nematode taxa served as a starting point for identification of the most conserved regions, which were subsequently used for design of various variants of forward and reverse primers. GenBank accession numbers of nematode mtCOI sequences used in the alignment are listed in the Supplementary Table 1 (Table S1). Primer combinations that worked best for genus *Helicotylenchus* and resulted in a successful amplification of partial mtCOI sequences are listed in Table 1. It is worth mentioning that, in their recent work, Palomares-Rius et al. (2018) used a primer pair originally developed by Kanzaki and Futai (2002), which allowed them to amplify approximately 660 bp, covering a broader region of this gene from *H. oleae*. Remarkably, the use of the hereby presented M3.5F and M8aR primer combination targets a mtCOI region of comparable length of 670, slightly shifted to the 3′ end.

**Table 1.** Overview of PCR primers designed in this study, which were used for mtCOI amplification from three *Helicotylenchus* spp. and one *Rotylenchus* sp.

Forward primer (5′-3′)	Reverse primer (5′-3′)	Approximate amplicon size	Name of species and corresponding GenBank sequence numbers
M3.5F: GGAGTGGiACARGiTGAAC	M8aR^a^: GCAACiACATAATAAGWATCATG	700	*H. pseudorobustus*: MG663105
			*R. uniformis*: MG663121
	M6.9R: ACCiACARTAAAiATATGATG	450	*H. pseudorobustus:* MG663104
			*H. varicaudatus:* MG663116; MG663116; MG663116
			*R. uniformis:* MG663122
M2F^b^: ATTGGiGSTTTTGGTAATT	RH1R: CCAACAATGAATATATGATG	600	*H. canadensis*: MG663099; MG663100
			*H. pseudorobustu*s: MG663106; MG663107; MG663109; MG663110; MG663111; MG663112; MG663113
			*H. varicaudatus*: MG663115
RH2F: GGTGGAAGAATTAATTTYTG		350	*H. canadensis*: MG663098; MG663101
			*H. varicaudatus*: MG663114; MG663118; MG663120

Note: i = inosine. ^a^This primer is a modified version of the COIR primer proposed by He et al. (2005). The primer was elongated by 4 nucleotides and two nucleotide modifications were incorporated; ^b^This primer is a shortened and modified version of the COI-F1 primer proposed by Kanzaki and Futai (2002). The primer was shortened from 29 nt to 19 and two nucleotide modification were introduced.

28S rDNA fragments were acquired using MCB1F and MCB1R primers (Dobosz et al., 2013). All primers used in this study were supplied either by Oligo.pl (Warsaw, PL) or by Sigma-Aldrich (USA). The amplification of the 28S rDNA and mtCOI fragments was performed in reactions containing 12.5 μl JumpStart Taq Ready Mix or RedTaq Ready Mix (Sigma-Aldrich), 1 μl of corresponding primer (5 μM), 1 to 3 μl DNA template and H_2_O with a total volume of 25 μl. Annealing temperatures used for the amplification of 28S rDNA and mtCOI fragments were 58 to 60°C and 40 to 43°C, respectively. All PCR reactions were performed in Veriti 96*-*Well Thermal Cycler (Applied Biosystems, Foster City, CA, USA). Amplicons were visualized by UV illumination after Midori Green (Nippon Genetics Europe, Duren, Germany) gel staining and gel electrophoresis. Excess dNTPs and unincorporated primers were removed from the PCR product using the Clean-Up Purification Kit (A&A Biotechnology, Gdynia, Poland). As a final step, the purified DNA was eluted in 40 μl H_2_O. Sequencing PCR reactions consisted of 1 μl BigDye Terminator v. 3.1 Ready Reaction Mix (ThermoFisher Scientific, Waltham, MA, USA), 2 μl BigDye sequencing buffer (ThermoFisher Scientific), 1.6 μl (5 μM) forward or reverse primer, and H_2_O with 10 μl total volume. The thermal profile for sequencing reactions comprised of an initial denaturation step at 96°C for 1 min, followed by 25 cycles at 96°C for 10 s, 50°C for 5 s and 60°C for 105 s. 28S rDNA and mitochondrial sequences were sequenced with an ABI 3500xL genetic analyzer (Applied Biosystems).

### Phylogenetic analyses

Multiple sequence alignments and subsequent comparison of the newly acquired (Tables 1 and 2) and GenBank-derived *Helicotylenchus* spp. sequences were performed using ClustalW algorithm as implemented in the BioEdit program v. 7.2.5 (Hall, 1999). The final multiple-sequence alignments were 595 and 588 nucleotide sites long in the case of 28S rDNA and mtCOI data, respectively. For the 28S rDNA data set, two *Rotylenchus* and two *Hoplolaimus* species were selected as outgroup: *R. uniformis* (MG653537, acquired in this study), *R. magnus* (EU280789), *H. seinhorsti* (DQ328752) and *H. galeatus* (EU626787). *Heterodera glycines* (HM462017) and *R. uniformis* (MG663121-2; this study) sequences were used as outgroups in mtCOI data set. Substitution models were tested using the “FindModel,” an online implementation of MODELTEST (Posada and Crandall, 1998). A GTR + I + G substitution model was chosen for both 28S rDNA and mtCOI DNA data sets. The Bayesian phylogenetic trees were constructed with the program MrBayes v. 3.1 (Ronquist and Huelsenbeck, 2003). In both cases, two independent runs were performed with four Markov chains per run. In case of the 28S rDNA, the program was run for 3,000,000 generations and in case of mtCOI for 1,000,000 generations, with a sampling frequency of 100 generations. Burn-in trees of 50,000 and 25,000 generations, respectively, were discarded. For the mtCOI data, the partition by codon position was set. The sampled trees from each run were combined in a single 50% majority-rule tree. The stabilization of the likelihood and parameters was checked with the program Tracer v. 1.6 (Rambaut et al., 2014).

**Table 2.** Hoplolaimidae species included in phylogenetic analyses.

Species	Individual	Soil sample code	Sample locality (Voivodeship)	Coordinates	Vegetation type	28S rDNA GenBank number	mtCOI GenBank number
*Helicotylenchus*	1	CH 0040/04	Dobrzyca (West Pomeranian)	N 54.172277 E 15.926119	*Buxus sempervirens L.;* nursery	MG653526	MG663098
*canadensis*	2	CH 0197/01	Ligota Mała (Lower Silesian)	N 51.126219 E 17.346800	*Rosa L.;* cultivation	–	MG663099
	3	CH 0199/01	Kąty Bystrzyckie (Lower Silesian)	N 50.312597 E 16.840489	*Rosa L.;* cultivation	MG653526	MG663100
	4	CH 0199/01	Kąty Bystrzyckie (Lower Silesian)	N 50.312597 E 16.840489	*Rosa L.;* cultivation	MG653527	–
	5	CH 0199/01	Kąty Bystrzyckie (Lower Silesian)	N 50.312597 E 16.840489	*Rosa L.;* cultivation	–	MG663101
	6	CH 0199/01	Kąty Bystrzyckie (Lower Silesian)	N 50.312597 E 16.840489	*Rosa L.;* cultivation	MG653526	–
*Helicotylenchus*	1	KW 0014/05	Sierpówko (Greater Poland)	N 52.473777 E 16.585961	Mixed forest	MG653532	MG663104
*pseudorobustus*	2	KW 0063/04	Brzostów (Greater Poland)	N 51.978670 E 17.405130	Mixed forest	MG653533	MG663104
	3	KW 0063/04	Brzostów (Greater Poland)	N 51.978670 E 17.405130	Mixed forest	MG653533	MG663105
	4	KW 0063/04	Brzostów (Greater Poland)	N 51.978670 E 17.405130	Mixed forest	–	MG663106
	5	KW 0008/01	Kleszczele (Podlaskie)	N 52.563534 E 23.312296	*Solanum tuberosum L.;* cultivation	MG653534	MG663107
	6	KW 0008/01	Kleszczele (Podlaskie)	N 52.563534 E 23.312296	*Solanum tuberosum L.;* cultivation	MG653532	MG663108
	7	KW 0008/01	Kleszczele (Podlaskie)	N 52.563534 E 23.312296	*Solanum tuberosum L.;* cultivation	–	MG663109
	8	KW 0154/01/02	Nowy Duninów (Masovian)	N 52.577483 E 19.502000	*Acer negundo L.;* fallow	–	MG663110
	9	KW 0154/01/02	Nowy Duninów (Masovian)	N 52.577483 E 19.502000	*Acer negundo L.;* fallow	MG653532	MG663111
	10	KW 0154/01/02	Nowy Duninów (Masovian)	N 52.577483 E 19.502000	*Acer negundo L.;* fallow	–	MG663112
	11	KW 0078/01	Radomierz (Lower Silesian)	N 50.909560 E 15.911490	Poaceae (R. Br.) Barnh.; meadow	MG653534	–
	12	KW 0078/01	Radomierz (Lower Silesian)	N 50.909560 E 15.911490	Poaceae (R. Br.) Barnh.; meadow	MG653533	MG663113
	13	KW 0080/02	Rybnica (Lower Silesian)	N 50.908020 E 15.675000	Fagopyrum Mill; cultivation	MG653532	–
*Helicotylenchus*	1	KW 0013/02	Turew (Greater Poland)	N 52.060160 E 16.819668	*Tilia* L.; park	–	MG663114
*varicaudatus*	2	KW 0013/01	Turew (Greater Poland)	N 52.060160 E 16.819668	*Platanus* L.; park	–	MG663115
	3	KW 0154/01/01	Nowy Duninów and Stary Duninów (Masovian)	N 52.577483 E 19.502000	*Acer negundo* L.; fallow	MG653535	–
	4	KW 0154/02	Nowy Duninów and Stary Duninów (Masovian)	N 52.577483 E 19.502000	*Acer negundo* L.; fallow	MG653535	MG663116
	5	KW 0154/02	Nowy Duninów and Stary Duninów (Masovian)	N 52.577483 E 19.502000	*Acer negundo* L.; fallow	MG653535	MG663117
	6	KW 0154/02	Nowy Duninów and Stary Duninów (Masovian)	N 52.577483 E 19.502000	*Acer negundo* L.; fallow	–	MG663118
	7	KW 0154/02	Nowy Duninów and Stary Duninów (Masovian)	N 52.577483 E 19.502000	*Acer negundo* L.; fallow	–	MG663119
	8	KW 0154/02	Nowy Duninów and Stary Duninów (Masovian)	N 52.577483 E 19.502000	*Acer negundo* L.; fallow	MG653535	MG663119
	9	KW 0154/01/02	Nowy Duninów and Stary Duninów (Masovian)	N 52.577483 E 19.502000	*Acer negundo* L.; fallow	–	MG663120
*Rotylenchus*	1	KW 0084/01	Czernia (Lubusz)	N 51.534330 E 15.240710	*Secale* L.; cultivation	MG653536	–
*uniformis*	2	KW 0088/01	Miodnica and Gorzupia (Lubusz)	N 51.708180 E 15.288050	*Solanum tuberosum* L.; cultivation	MG653537	–
	3	KW 0088/01	Miodnica and Gorzupia (Lubusz)	N 51.708180 E 15.288050	*Solanum tuberosum* L.; cultivation	MG653536	–
	4	KW 0088/01	Miodnica and Gorzupia (Lubusz)	N 51.708180 E 15.288050	*Solanum tuberosum* L.; cultivation	MG653538	MG663121
	5	KW 0088/01	Miodnica and Gorzupia (Lubusz)	N 51.708180 E 15.288050	*Solanum tuberosum* L.; cultivation	MG653539	–
	6	KW 0067/01	Toruń (Kuyavian-Pomeranian)	N 53.027500 E 18.595470	lawn	MG653540	MG663122

Note: Individuals from whom both 28S rDNA and the mtCOI sequences were obtained are marked in grey.

## Results

**Figure 1:**
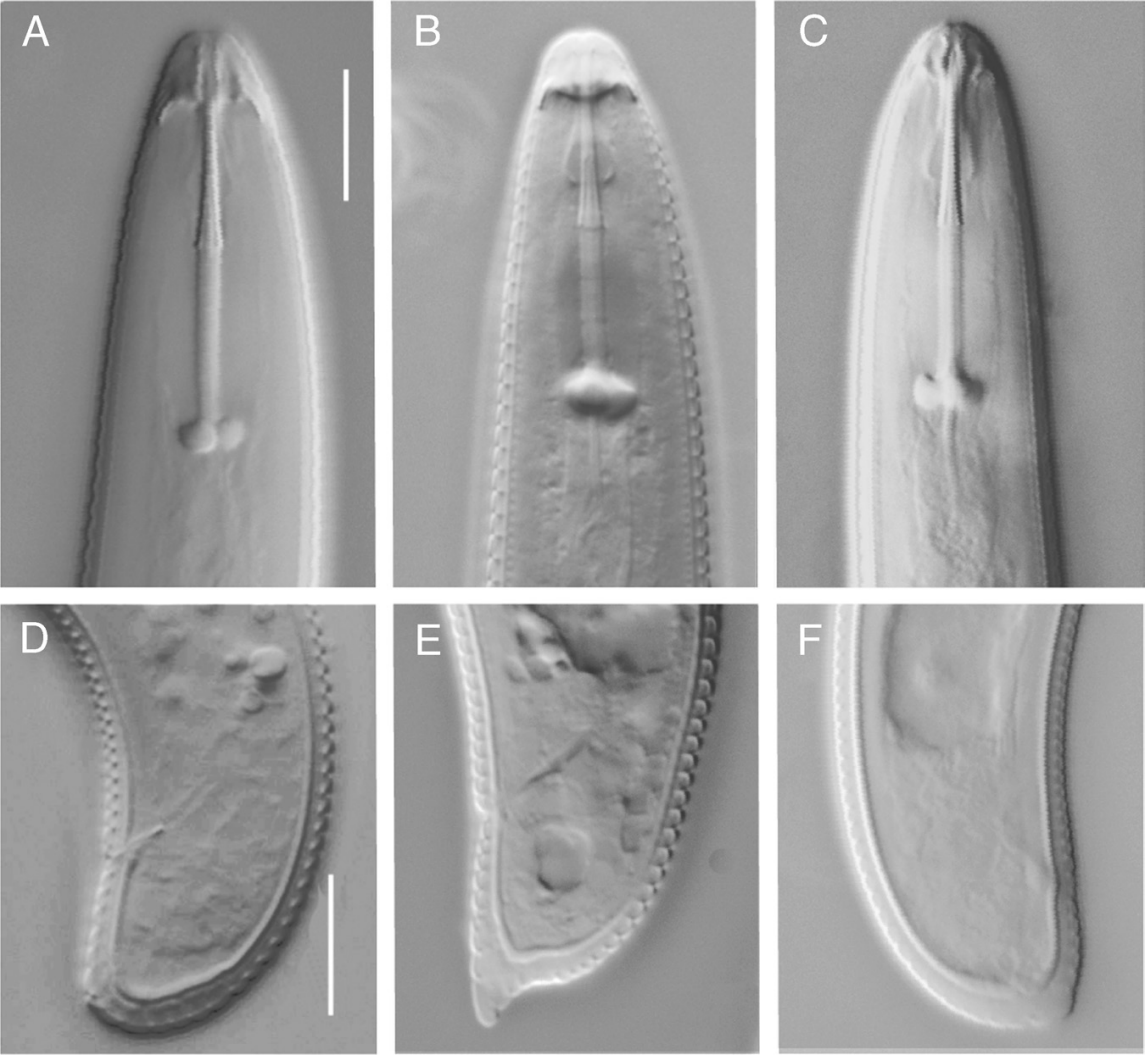
Anterior and posterior regions of three *Helicotylenchus* species: (A) Anterior body and (D) tail of *H. canadensis* (CH 0199/01). (B) Anterior body and (E) tail of *H. pseudorobustus* (KW 0154/01). (C) Anterior body and (F) tail of *H. varicaudatus* female (KW 0154/02). (Scale bar =10 μm).

*Helicotylenchus canadensis* (Waseem, 1961).

(Fig. 1A and D; Table 3).

**Table 3.** Morphometrics of *Helicotylenchus canadensis* populations from different localities.

Locality	Populations analyzed in this study, Poland	Holotype, Quebec, Canada *acc.* (Waseem, 1961)	Paratypes, Quebec, Canada *acc.* (Waseem, 1961)	Rothamsted, England *acc.* Yuen, 1964	Populations from New Zealand, *acc.* (Yeates and Wouts, 1992)	Populations from temperate Europe, *acc.* (Brzeski, 1998)
*n*	5		15	20	25	
L	793.1 ± 54 (698.7–866.6)	860	780 (680–970)	680–840	726–906	680–1040
a	22.4 ± 1.16 (20.9–24.1)	24.5	24.3 (20.0–30.4)	18–26	23–31	20–31
b	5.9 ± 0.34 (5.3–6.3)	5.2	5.4 (4.8–6.7)	5.3–6.2	5.4–7.9	5.3–8.1
c	50.5 ± 8.1 (38.5–61.8)	62.3	56.4 (48.7–65.0)	36–54	45–63	36–72
c′	0.9 ± 0.1 (0.8–1.1)	0.9–1.4	–	–	0.7–1.1	0.6–1.0
V	58.3 ± 1.9 (55.5–59.5)	64	64(61–66)	59–64	57–63	58–66
Stylet length	28.8 ± 0.94 (28.2–30.7)	30	30 (28–30)	31–33	28–33	27–33.5
Pharyngal length	135.1 ± 11.6 (120.2–152.7)	–	–	–	150–175	103–140
Max. body diam.^a^	35.4 ± 2.9 (30.3–38.9)	–	–	–	26–37	–
Tail length	16.2 ± 3.1 (11.3–20.5)	–	12–16	15–22	13–19	12–22
Anal body diameter	17.9 ± 2.34 (13.3–20.3)	–	–	–	–	–
Tail annuli number	13.2 ± 2.5 (10.0–16.0)	–	–	8–12	8–12	6–12
Phasmid position (number of annules anterior to anus)	5.0±2.7 (3.0-9.0)	–	–	4–9	6–12	3–12

Note: ^a^Measured at vulval region. Measurements are given in μm, V in %, and in the form: mean ± s.d. (range).

The morphological characteristics of *H. canadensis* females (*n* = 5) are as follows: the body is spiral when relaxed. The lateral field exists with four lines. Head is conical, truncated, bearing four to five annuli, continuous with body contour. Pharyngeal glands overlap intestine on the ventral side. The median oesophagus bulb is oval. Spermatheca is empty. Phasmids’ position is three to nine annuli anterior to anus. The tail is slightly curved dorsally, bearing 10 to 16 annuli, a bit ventrally flattened with rounded, striated terminus.

Remarks: The morphological characteristics of *H. canadensis* under study agree with the first description of the species (Waseem, 1961) as well as following ones given for populations from England (Yuen, 1964), New Zealand (Yeates and Wouts, 1992), Poland (Brzeski, 1998), and Hungary (Andrássy, 2007). Although the ranges of some parameters (V, pharyngeal length, body diameter, tail length, and phasmids’ position) slightly differed from those given for the paratypes, they corresponded well to the ranges given by Brzeski (1998) for *H. candensis* occurring in European regions of the temperate climate.

Males: Not found.

Molecular data: 28S rDNA: MG653526-MG653527, mtCOI: MG663098-MG663101.

Locality and habitat: See Table 2.

*Helicotylenchus pseudorobustus* (Steiner, 1914; Golden, 1956).

(Fig. 1B and E; Table 4).

**Table 4.** Morphometrics of *Helicotylenchus pseudorobustus* populations from different localities.

Locality	Populations analyzed in this study, Poland	Topotypes, Switzerland *acc.* (Sher, 1966)	Topotypes, Switzerland *acc.* (Fortuner et al., 1984)	Populations from New Zealand *acc.* (Yeates and Wouts, 1992)	Populations from temperate Europe *acc.* (Brzeski, 1998)	Populations from California, USA *acc.* (Subbotin et al., 2015)	Populations from Iran *acc.* (Shokoohi et al., 2018)
*n*	13	20	20	86		25	22
L	767.2 ± 81.3 (675.1–865.9)	600–820	764	697–840	560–820	642–895	666–934
a	26.4 ± 4.7 (21.7–34.3)	27–34	28	27.5–34.9	24–34	25.3–31.8	24–35
b	6.0 ± 0.9 (5.8–8.1)	6.0–7.2	–	5.0–8.1	4.2–8.6	5.1–7.3	4.2–6.6
c	42.6 ± 12.2 (34.1–64.0)	32–52	48.4	33–61	32–52	31.2–46.9	32.6–59
c′	1.0 ± 0.3 (0.6–1.5)	0.9–1.4	–	0.9–1.5	0.8–1.4	1.0–1.4	1–3.2
V	61.9 ± 5.5 (48.1–71.8)	59–64	61.6	59–66	59–67	58.4–64.6	46–65
Stylet length	28.0 ± 0.7 (26.5–29.5)	26–30	27.1	22–28	24–30.5	25–27.5	23–27
Pharyngal length	124.9 ± 21.3 (102.7–173.4)	–	116	133–178	104–128	116–160	120–148
Max. body diam.^a^	29.1 ± 4.9 (21.9–35.5)	–	27.8	23.7–28.5	–	25–31	24–31
Tail length	17.3 ± 3.3 (12.5–23.20)	–	15.9	14.6–19.5	15–22	16–24	13.7–24.5
Anal body diam.	13.9 ± 2.2 (12.2–19.7)	–	15.6	–	–	15–20	13.7–16
Tail annuli number	10.0 ± 2.4 (6.0–13.0)	7–12	–	–	7–17	8–15	–
Phasmid position (number of annules anterior to anus)	7.5 ± 2.3 (4–10)	2–7	3–11	6–11	2–12	5–10	–

Note: ^a^Measured at vulval region. Measurements are given in μm, V in %, and in the form: mean ± s.d. (range).

The morphological features of the *H. pseudorobustus* females (*n* = 13) are as follows: the body is spiral when relaxed. The lateral field exists with four lines. The lip region is hemispherical, continuous with body contour, with four to five annuli. Stylet is present with anteriorly flattened knobs. Pharyngeal glands overlap intestine on ventral side; the median pharyngeal bulb is oval. Spermatheca is empty. Phasmids’ position is seven to ten annules anterior to anus. The tail is curved dorsally, bearing 6 to 13 annuli, with small, but distinct rounded ventral projection.

Remarks: The morphological features of the investigated *H. pseudorobustus* individuals were congruent with the descriptions of the topotypes (Sher, 1966; Fortuner, 1984). However, the range of most of the parameters was larger. *H. pseudorobustus* is a cosmopolitan species and there are many descriptions available of this species from different regions. Consequently, the ranges of many parameters extended, compared to the original ones. Most morphometric parameters of *H. pseudorobustus* analyzed in our study were consistent with those given by Yeates and Wouts (1992), Brzeski (1998), Subbotin et al. (2015), and Shokoohi et al. (2018). However, lower ranges of c′, tail length and anal body diameter were slightly smaller (Table 4). This finding confirms the claims of Fortuner et al. (1984) and Subbotin et al. (2015) concerning the large morphological variability of *H. pseudorobustus.*

Males: Not found.

Molecular data: 28S rDNA: MG653532-MG653534, mtCOI: MG663104-MG663113.

Locality and habitat: See Table 2.

**Figure 2:**
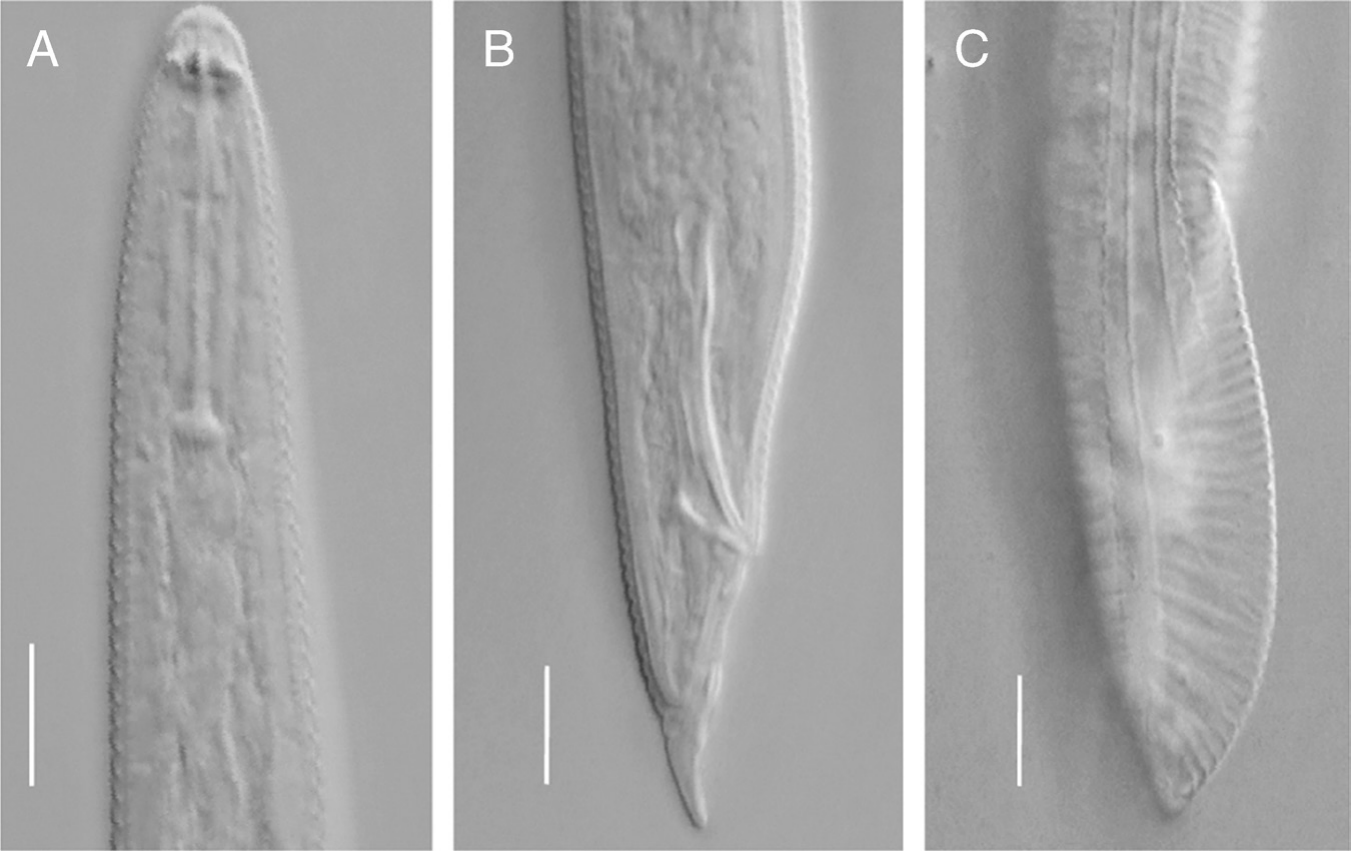
Anterior and posterior regions of *H. varicaudatus* male (KW 0154/02). (A) Anterior body. (B) Tail, with focus on spicule and gubernaculum. (C) Tail, with focus on bursa and phasmid. Images of male were captured from the video documentation of temporary slides.

*Helicotylenchus varicaudatus* (Yuen, 1964).

(Fig. 1C and F; Fig. 2A to C; Table 5).

**Table 5.** Morphometrics of *Helicotylenchus varicaudatus* populations from different localities.

Locality	Populations analyzed in this study, Poland	Holotype, Rothamsted, England, *acc.* (Yuen, 1964)	Paratypes, Rothamsted, England, *acc.* (Yuen, 1964)	Populations from New Zealand *acc.* (Yeates and Wouts, 1992)	Populations from temperate Europe *acc.* (Brzeski, 1998)	Population from Portugal *acc.* (Schreck Reis et al., 2010)	Populations analyzed in this study, Poland	Population from Portugal *acc.* (Schreck Reis et al., 2010)
*n*	5 females		19 females	48 females		40 females	4 males	10 males
L	734.7 ± 101.1 (623.8–876.5)	670	580–670	586–814	520–790	510–890	676.3 ± 39.1 (612.8–715.2)	530–700
a	24.9 ± 0.9 (24.8–26.0)	22	18–26	22–32	18–29	23.5–35.8	25.0 ± 2.4 (22.5–27.8)	30.3–37.4
b	5.5 ± 1.2 (4.2–7.2)	4.8	4.3–5.2	4.9–7.7	4.3–7.5	5.8–8.7	6.7 ± 0.7 (5.7–7.2)	6.6–8.5
c	42.5 ± 7.3 (34.1–52.4)	–	39–50	36–77	38–75	39.4–70	33.5 ± 2.2 (31.4–37.2)	34–37.3
c′	1.3 ± 0.5 (0.7–1.8)	–	–	0.6–1	0.5–1.2	0.7–1.3	1.7 ± 0.1 (1.6–1.9)	1.6–2.2
V	62.5 ± 1.9 (60.1–65)	62	60–63	59–67	59–66	61–67		
Stylet length	29.7 ± 1.2 (29.0–31.3)	32	29–33	31–33	25–33	22–26	26.3 ± 0.7 (25.2–27.1)	20–23
Pharyngal length	131.9 ± 7.0 (120.7–139.9)	–	–	104–136	99–113	120–167	113.8 ± 15.0 (99.3–134.5)	126–172
Max. body diam.^a^	31.3 ± 6.5 (24.0–40.3)	–	–	22–34	–	14–26	25.9 ± 3.0 (24.6–30.7)	17–20
Tail length	17.8 ± 4.1 (13.3–21.4)	–	12–17	8–19	8–19	9.5–17.5	20.3 ± 1.1 (19.2–22.0)	17–20
Anal body diam.	14.8 ± 2.8 (12.0–15.4)	–	–	–	–	10–17	11.9 ± 1.1 (10.4–13.6)	9–11
Tail annuli number	5.8 ± 1.3 (4–7)	–	6–11	6–12	4–14	4–8	–	8–11
Phasmid position (number of annules anterior to anus)	2.0 ± 2 (0–5)	–	–	−1–+5	−3–+3	−1–+4	–	−4–+7
Spicula length							28.1 ± 1.9 (26.5–30.7)	20–25
Gubernaculum							9.1 ± 0.9 (7.9–10.2)	4.4–7.0

Note: ^a^Females measured at vulval region, males measured at mid-body. Measurements are given in μm, V in %, and in the form: mean ± s.d. (range).

The morphological features of the *H. varicaudatus* females are as follows: (*n* = 5). Habitus is loose spiral, the lateral field exists with four lines, head is conical, with four to five annuli, continuous with body contour. The length of stylet is 29 to 31.3 μm, esophageal glands overlap intestine on ventral side. Spermatheca is empty. The tail of specimens is dorsally convex with a nearly straight ventral contour. The connection of dorsal contour with ventral one is almost at right angle. The tail is irregular at ventral terminus, with four to seven annuli and phasmids are located near anus (at anus level up to five annuli anterior to anus).

Remarks: Two *H. varicaudatus* populations were analyzed: one with and one without males (from Nowy Duninów/ Stary Duninów and Turew, respectively). Although, morphologically, our individuals corresponded well with the first description of *H. varicaudatus* made by Yuen (1964), ranges of most of the parameters differed from the original ones. *H. varicaudatus* was recorded in many countries and more descriptions of this species are available (Yeates and Wouts, 1992; Brzeski, 1998; Schreck Reis et al., 2010). Most parameters obtained in the current study were congruent with these descriptions. Nevertheless, tails of some females analyzed in our study were a little longer than tails of *H. varicaudatus* females described by other authors. It substantiates the claims of many authors (Yuen, 1964; Loof (1984); Brzeski, 1998) on the large variability of tail shape in this species and relates to the origin of the species name. Strikingly, female spermathecae of the bisexual population analyzed in our study were empty. Analyzing Dutch, bisexual population of *H. varicaudatus*, Loof (1984) also observed that some females had empty spermatheca. Of 86 analyzed females, only 47 had spermatheca filled with sperm.

The morphological features of the *H. varicaudatus* males (*n* = 4) are as follows: arcuate ventrally, in many respects (number of lines in lateral field, ventral overlapping of oesophageal glands, shape of head, number of head annuli) similar to females. The body is usually shorter than females (612.8 to 715.2 vs 623.8 to 876.55), with shorter stylet than females (25.2 to 27.1 vs 29.0 to 31.3). Bursa is extended to the end of tail. Spicule is slightly arcuate 26.5 to 30.7 µm, gubernaculum 7.9 to 10.2 µm.

Remarks: Although females of *H. varicaudatus* have been recorded in many countries, males of this species occur extremely rarely. They were noted only in some locations in the Netherlands (Loof, 1984; Bongers, 1994), in Poland (Brzeski, 1998) and in coastal sand dunes in Portugal (Schreck Reis et al., 2010). Therefore, the descriptions of *H. varicaudatus* males are very scarce. In our study, we found only four males of *H. varicaudatus* in the rhizosphere of ash-leaved maple. Although these specimens corresponded to the description of Loof (1984) in respect of morphology, they differed in some morphometric parameters in relation to the Portuguese population. The males analyzed in our study had longer spicules than in the Portuguese population, 26.5 to 30.7 µm vs 20 to 25 µm. However, the spicule length of *H. varicaudatus* males herein did not significantly differ from the spicule length (23 to 28 µm) of males from Poland, as previously described by Brzeski (1998). The gubernaculum of *H. varicaudatus* males obtained in this study was much longer than those of males from Portugal as well as those from Poland (7.9 to 10.2 µm vs 5 to 7 µm).

Molecular data: 28S rDNA: MG653535, mtCOI: MG663114-MG663120.

Locality and habitat: See Table 2.

### Sequence analysis

All sequences reported in this study have been deposited in GenBank and their accession numbers are listed in Table 2. By the use of the newly designed primers, we obtained mitochondrial sequences from 4 representatives of *H. canadensis*, 11 of *H. pseudorobustus* and 8 of *H. varicaudatus* (Table 2). The particular amplicon and a derived size of the newly obtained sequences varied depending on the primer combination used (Table 1). The designed primers, in most combinations, targeted the region relatively closer to the 5′ end of the mtCOI gene sequence. The longest PCR amplicons, which were approximately 700 bp, were obtained using the M3.5 F and M8aR primer combination, which also amplified a part of the mitochondrial sequence closer to the 3′ end of the mtCOI gene. However, the most successful primer combination was the M2F and RH1R one, which allowed for the amplification of PCR product of approximately 600 bp from all analyzed *Helicotylenchus* species.

**Figure 3:**
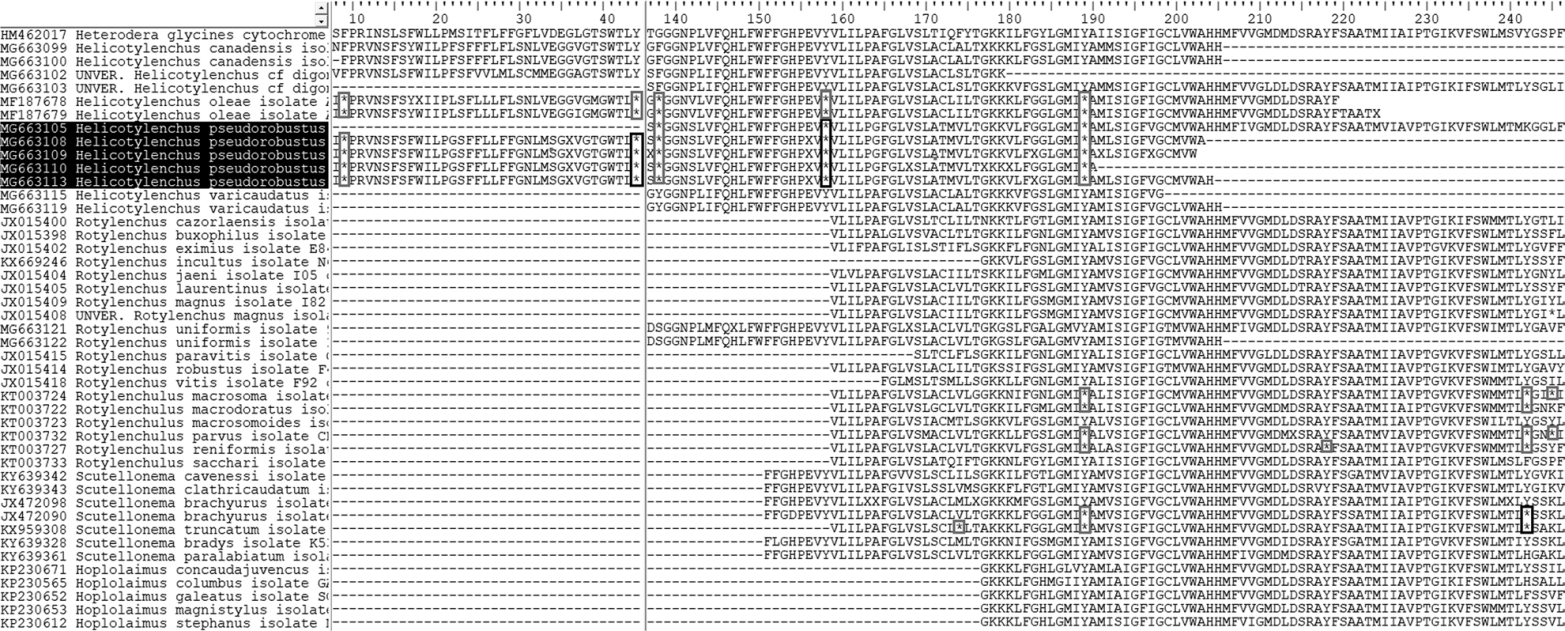
The alignment of partial mtCOI sequences from representatives of Hoplolaimidae, translated from nucleotide into amino acid sequences using the standard invertebrate mitochondrial genetic code. X, unknown amino acid; *, TAA (grey frame) or TAG (black frame) codon.

There was no intra-specific nucleotide variation concerning the four mtCOI sequences derived from *H. canadensis*. The potential minor nucleotide differences concerned few degenerate nucleotides, namely, K (G or T), Y (C or T), and R (A or G). The highest amplification success was obtained for the *H. pseudorobustus* and *H. varicaudatus*. By the use of three of the four presented primer combinations, we were able to obtain amplicons and subsequent mitochondrial sequences from 11 and 8 nematode individuals, respectively. The variability of the sequence amplified from *H. pseudorobustus* involved only some minor ambiguous nucleotides, possibly due to sequencing error. The only significant nucleotide substitution (A/T) was localized in the middle of the sequence at site 418 (MG663108), which had no influence on the potential amino acid sequence translation. Importantly, the more insightful analysis of the *H. pseudorobustus* mitochondrial nucleotide sequences revealed that all of them contain multiple TAA and TAG codons, which in the regular invertebrate mitochondrial genetic code would be translated as termination codons. A comparison of these sequences with the publicly available sequences derived from other Hoplolaimidae species (Fig. 3) suggests that Tyrosine (Y) and Phenylalanine (F) codons are the most vulnerable to such substitutions in this family, and that *H. oleae*-derived sequences also contain TAA codons (Cantalapiedra-Navarrete et al., 2013; van den Berg et al., 2016; Palomares-Rius et al., 2018). Both TAA and TAG triplets were also detected in sequences derived from two *Scutellonema*: *S. brachyurus* and *S. truncatum*, reported from study of van den Berg et al. (2013, 2017). No amino acid sequence ambiguities were predicted for the two other *Helicotylenchus* species analyzed here. Furthermore, no intra-specific variation on the nucleotide level was observed in the case of mtCOI sequences obtained from *H. varicaudatus* individuals.

28S rDNA fragments were successfully acquired from four *H. canadensis*, nine *H. pseudorobustus*, and four *H. varicaudatus* individuals (Table 2). All fragments were approximately 550 nucleotides long and they were characterized by a lack of intra-specific sequence variation.

The highest values of inter-specific variations were observed between *H. pseudorobustus* and two other species: 32 to 39% in case of mitochondrial sequences and 12 to 13% in case of the 28S rDNA. The lowest inter-specific variation was between *H. canadensis* and *H. varicaudatus*, which was on the level of 16 to 20% in the case of mitochondrial sequences and 5% in the case of 28S rDNA.

### 28S rDNA and mtCOI-based phylogenetic relationships of the investigated Hoplolaimidae species

**Figure 4:**
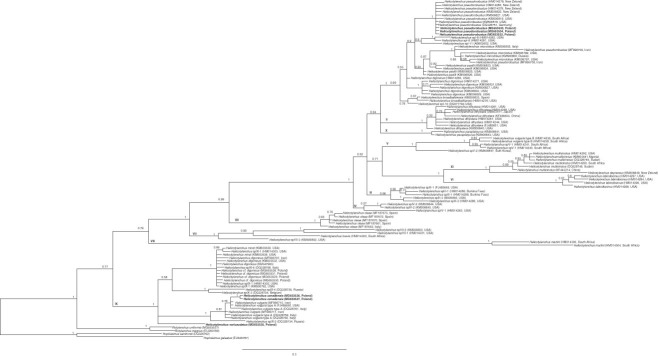
Phylogeny of the genus *Helicotylenchus*, as inferred by Bayesian analysis of 28S rDNA. The numbers near nodes indicate posterior probabilities. Roman numerals indicate major clades following Subbotin et al. (2011, 2015). The original 28S rDNA sequences are in boldface font.

Bayesian phylogenetic analysis of the 28S rDNA sequences derived from various *Helicotylenchus* species resulted in a tree topology (Fig. 4) that agrees with the phylogenetic data presented by Subbotin et al. (2015), Palomares-Rius et al. (2018), and Shokoohi et al. (2018). In total, 12 *Helicotylenchus* clades (I to XI, as defined in Subbotin et al. 2015; number XII was given in this study to the *H. oleae* lineage) were recovered, and the *Helicotylenchus* sequences acquired here were inferred to belong to two of them. According to the expectations, *H. pseudorobustus* sequences were placed in Clade I and clustered with the other publicly available *H. pseudorobustus* sequences. The rest of the new sequences derived from the two other analyzed *Helicotylenchus* species were inferred to be in Clade IX. However, the representatives of each of the two species were redistributed as lineages separated by closer relationships to other species. Sequences of *H. canadensis* clustered within Clade IX, together with representatives of *H. vulgaris* Type A, whereas the *H. varicaudatus*-derived sequence was inferred to be a sister lineage of all other Clade IX taxa. Designating *Hoplolaimus* species as outgroup, *Rotylenchus uniformis* (MG653537, obtained in this study), together with its congener *R. magnus* (EU280789), formed the sister group to *Helicotylenchus*.

**Figure 5:**
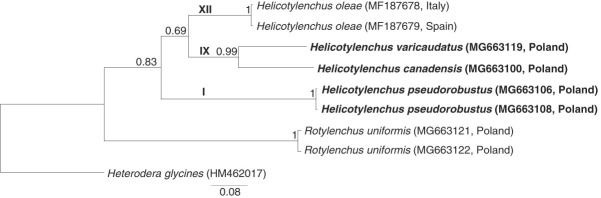
Phylogeny of the genus *Helicotylenchus*, as inferred by Baysian analysis of partial mtCOI sequences. Roman numerals indicate major clades according to Subbotin et al. (2011, 2015). The original mtCOI sequences are in boldface font.

Congruent with results of 28S rRNA analyses above, the topology of the phylogenetic tree inferred from mtCOI showed that *H. canadensis* and *H. varicaudatus* share the closest pairwise relationship of the three *Helicotylenchus* species analyzed (Fig. 5) and they were inferred to be in Clade IX of the 28S rDNA-based tree. The species *H. pseudorobustus* (representative of Clade I) represented a separate lineage, whose most recent common ancestor with the former two species was that of all *Helicotylenchus* species. However, the relationships between the three distant lineages (Clades I, IX and XII) have not been resolved on the basis of the available mitochondrial data, most probably due to insufficient number of analyzed taxa.

## Discussion

Morphological and molecular data in this study included three *Helicotylenchus* species: *H. canadensis, H. pseudorobustus* and *H. varicaudatus*, extracted from various locations and vegetation types in Poland (Table 2). The morphological evaluation of the *H. pseudorobustus* nematode individuals and their positioning in the Clade I of the *Helicotylenchus* 28S rDNA tree agreed with the type characterization of this species, as described in Subbotin et al. (2015). Newly generated 28S rDNA sequences from *H. canadensis* and *H. varicaudatus* that are positioned in Clade IX are reported for the first time for those species. Morphological and morphometrical studies on these species were congruent with previous descriptions. The inferred, close phylogenetic relationship of *H. canadensis* to *Helicotylenchus vulgaris* (Yuen, 1964) supports morphological comparisons of these two species as being very similar. The most clearly observable differences between these two species were the length of the tail and the width of the annuli at the tail end: the tail of *H. vulgaris* is shorter than the tail of *H. canadensis*, and the middle annuli at the end of the *H. vulgaris* tail are narrower than other annules, whereas the annuli of the *H. canadensis* tail are of equal width. The mtCOI-based phylogenetic analysis included four newly sequenced *Helicotylenchus* spp. (Fig. 5) and it also revealed that *H. canadensis* and *H. varicaudatus* are more closely related to each other than either is to *H. pseudorobustus*.

By providing the 28S rDNA and mtCOI data acquired in this study, the molecular characteristics of *H. canadensis, H. pseudorobustus* and *H. varicaudatus* were substantially enriched. So far, *H. canadensis* and *H. varicaudatus* had been characterized only by 18S rDNA fragments (van Megen et al., 2009; Schreck Reis et al., 2010). The 28S rDNA region is often more variable than the small subunit ribosomal DNA (Hillis and Dixon, 1991; Holterman, 2008) and it has been useful for resolving phylogenetic relationships within Hoplolaiminae (Bae et al., 2009; Subbotin et al., 2011, 2015; Palomares-Rius et al., 2018). Accordingly, the majority of the *Helicotylenchus* sequences deposited in GenBank comprise 28S rDNA data. Although GenBank already included 18S rDNA and 28S rDNA sequences for *H. pseudorobustus*, this study provides the first mtCOI fragments for this species. Characterized by a very high sequence variability, the mitochondrial data of *Helicotylenchus* species as well as other nematode genera are slowly increasing, being particularly useful in supporting the process of delineation of the most dubiously designated species.

The mitochondrial DNA region encoding for mtCOI gene has been widely explored for barcoding application purposes in many animal organisms (Rach et al., 2017). It was also used in Hoplolaimidae and other nematode groups (Kanzaki and Futai, 2002; Derycke et al., 2010; Riepsamen et al., 2011; Gutiérrez-Gutiérrez et al., 2012; Kumari and Subbotin, 2012; van den Berg et al., 2013; Cantalapiedra-Navarrete et al., 2013; Holguin et al., 2015; Vovlas et al., 2015; van den Berg et al., 2017; Sanchez-Monge et al., 2017; Skwiercz et al., 2017). In this study, to enhance the process of delineation and species identification within genus *Helicotylenchus*, which was underrepresented in terms of available mtCOI sequences, a new set of mtCOI primers was developed and tested. These newly designed primers offer an alternative to the most frequently used mtCOI primers combinations, especially in a situation when the original ones fail in the amplification of a desired PCR product. Most of the primer combinations presented in this publication allowed for the amplification of the fragments closer to the 5′ end of the assumed cytochrome oxidase c nucleotide sequence. The primer M2F in combination with the RH1R primer was the most successful in our study and allowed for amplification of a mtCOI sequence (600 bp) from all *Helicotylenchus* species analyzed here. The longest mtCOI sequence, including more nucleotides at its 3′ end, was acquired using the M3.5F and M8aR primer combination on *H. pseudorbustus* (and *R. uniformis* that was used as an outgroup species).

The inspection of mitochondrial sequences obtained here shows that all 11 mtCOI sequences from the nematode representatives of *H. pseudorobustus* were almost identical and included TAA and TAG codons at the same implied amino acid positions. Strikingly, such sequences were obtained each time for the *H. pseudorobustus* representatives, using three different mtCOI-targeted primer combinations (Table 1), whereas none of the sequences acquired from the other two *Helicotylenchus* species analyzed here revealed such nucleotide substitutions. In contrast, the homologous triplets in the mtCOI sequences of *H. candadensis* and *H. varicaudatus* instead contain triplets translated into tyrosine (TAT) and the phenylalanine (TTT) according to the standard invertebrate mitochondrial genetic code. This finding suggests alterations to the mitochondrial genetic code in *H. pseudorobustus*, if sequences are indeed translated through putative stop codons in this species.

Genetic code alterations in both nuclear and mitochondrial genomes have been reported in various organisms (Bessho et al., 1992; Telford et al. 2000; Lavrov et al., 2012; Lavrov, 2014; Bezerra et al., 2015; Pánek et al., 2017). In their studies, Jacob et al. (2009) showed that such alterations can also be found in nematodes. In the mitochondrial genome of nematode *Radopholus similis* (Pratylenchidae), the UAA (TAA) codon is reassigned from translation termination to tyrosine. Consequently, the usage of the TAA codon can be observed in mtCOI sequences acquired from various Hoplolaimidae species, including *Rotylenchulus* spp. (van den Berg et al., 2016) and the recently investigated *H. oleae* (Palomares-Rius et al., 2018). Although the simultaneous presence of TAA and TAG codons can be found in the sequences of *Scutellonema brachyurus* and *S. truncatum*, derived from the study of van den Berg et al. (2013, 2017), the authors did not elaborate on this matter.

On the contrary, the occurrence of stop codons in the mitochondrial sequences acquired from nematodes could also be an indication of nuclear copies of mitochondrial-derived genes (numts) or COI pseudogenes, as were highlighted by Holguin et al. (2015) in their studies on genetic diversity of *Hoplolaimus* spp. Although the researchers did not detect such changes in the mtCOI sequences in the species of their interest, they raised an important issue concerning the use of mtCOI sequences in the phylogenetic studies of nematodes. As long as the occurrence of TAG codons in the reported sequences is properly investigated, phylogenetic analyses based on mtCOI sequences should be treated with suitable consideration.

## Appendix

Supplementary Table S1 available upon request from the author.
